# The enzyme carbonic anhydrase as an integral component of biogenic Ca-carbonate formation in sponge spicules^☆^

**DOI:** 10.1016/j.fob.2013.08.004

**Published:** 2013-08-16

**Authors:** Werner E.G. Müller, Heinz C. Schröder, Ute Schlossmacher, Meik Neufurth, Werner Geurtsen, Michael Korzhev, Xiaohong Wang

**Affiliations:** a ERC Advanced Investigator Grant Research Group at Institute for Physiological Chemistry, University Medical Center of the Johannes Gutenberg University Mainz, Duesbergweg 6, Mainz D-55128, Germany; b Department of Conservative Dentistry, Periodontology and Preventive Dentistry, Hannover Medical School, Carl-Neuberg-Strasse 1, Hannover 30625, Germany

**Keywords:** Sponge, Calcareous spicules, Carbonic anhydrase, Crystal formation, *Sycon raphanus*

## Abstract

The inorganic scaffold of the spicules, the skeletal elements of the calcareous sponges, is formed of calcium carbonate (CaCO_3_). The growth of the approximately 300-μm large spicules, such as those of the calcareous sponge *Sycon raphanus* used in the present study, is a rapid process with a rate of about 65 μm/h. The formation of CaCO_3_ is predominantly carried out by the enzyme carbonic anhydrase (CA). The enzyme from the sponge *S. raphanus* was isolated and prepared by recombination. The CA-driven deposition of CaCO_3_ crystallites is dependent on temperature (optimal at 52 °C), the pH value of the reaction assay (7.5/8.0), and the substrate concentration (CO_2_ and Ca^2+^). During the initial phase of crystallite formation, ≈40 μm large round-shaped deposits are formed that remodel to larger prisms. These crystal-like prisms associate to each other and form either rope-/bundle-like aggregates or arrange perfectly with their smaller planes along opposing surfaces of the sponge spicule rays. The CA-dependent CaCO_3_ deposition can be inhibited by the CA-specific inhibitor acetazolamide. The Michaelis–Menten constant for the CA-driven mineralization has been determined to be around 8 mM with respect to CaCO_3_. The deposits formed have a Martens hardness of ≈5 GPa. The data presented here highlights for the first time that calcite deposition in the sponge system is decisively controlled enzymatically. This data will contribute to the development of new strategies applicable for the fabrication of novel biomaterials.

## Highlights

• Carbonic anhydrase (CA) catalyzes the reversible interconversion of CO_2_ and H_2_O to HCO_3_ (and protons).
• This enzyme is a major factor controlling and accelerating CaCO_3_ deposition *in vitro*.
• We examined the role of CA in calcareous spicule formation in the sponge *Sycon raphanus*
• We determined the parameters for enzymatically controlled CA-driven CaCO_3_ deposition.
• We outline the reactions in calcareous spicule formation, from abiogenic to biogenic CaCO_3_ synthesis.

## Graphical abstract

Bio-calcite: We learnt from nature (calcareous sponges) how large calcite structures are fabricated under physiological conditions. Here we outline that it is the enzyme carbonic anhydrase that has a crucial role on the bio-synthesis of Ca-carbonate. The kinetic parameters are given for the *in vitro* synthesis of Ca-carbonate.

**Figure:**
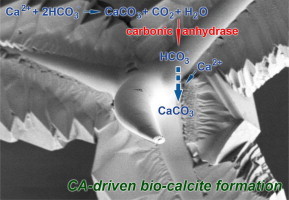

## 1 Introduction

Calcium carbonate [CaCO_3_] is a biomineral that constitutes the inorganic scaffold of the skeletal elements in non-metazoan (e.g. the single-celled algae coccolithophores) and also metazoan taxa [1,2]. The evolutionary oldest metazoans that invented Ca-carbonate as a scaffold for their skeleton are the calcareous sponges (Class Calcarea) that emerged on Earth, approximately 540 Ma [3]. While the first sponge taxa (Hexactinellida; Demospongia), as the earliest metazoans that diverged from the common metazoan ancestor, the Urmetazoa [4], comprise a siliceous skeleton, the calcareous sponges substitute the inorganic scaffold bio-silica for bio-calcite, very likely due to environmental constraints, the accumulation of Ca-carbonate in the ancient oceans [5]. Even though the present-day oceans are supersaturated with respect to CaCO_3_, only very rarely spontaneous abiotic precipitation is seen [6]. In biological systems, e.g. sponges, molluscs or echinoderms, Ca-carbonate is taken up from the aqueous environment as bicarbonate via specific membrane transporters [7] characterized by a Michaelis–Menten constant of around 50 mM [8]. At this concentration, Ca-carbonate precipitates at an extent of about 50% during an incubation period of 20 h in an ammonium carbonate diffusion/“dessicator assay” at a pH of 7–8 [9]. However, this reaction velocity is too slow to account for the observed Ca-carbonate deposition, measured *in vivo*, e.g. in the sponge spicule formation in *Sycon* sp. [10]; those spicules have, with a diameter of around 4 μm, a very fast growth rate of 65 μm/h. Since the calcitic deposition reaction is exergonic [11], an acceleration of the reaction velocity can be reached by lowering the activation energy either allowing the process of Ca-carbonate deposition to proceed on a functionalized organic surface (see Ref. [1]) or by coupling of the membrane-bound bicarbonate transporter with the soluble enzyme, the carbonic anhydrase [CA] [7]. While the accelerating or the decelerating proteinaceous components within biogenic Ca-carbonate skeletal structures, e.g. mollusk shell, have been extensively described (reviewed in Ref. [12]), an enzyme kinetic analysis of the CA during the Ca-carbonate deposition reaction has not been published. CAes form reversibly bicarbonate by hydration of carbon dioxide (CO_2_), a reaction which represents the rate-limiting step in the process of Ca-carbonate precipitation in the presence of Ca cations [13]. The CAes are among the fastest catalyzing enzymes and – in turn – represent a key catalyst in the fixation of CO_2_ during deposition of Ca-carbonate [14].

**Fig. 1:**
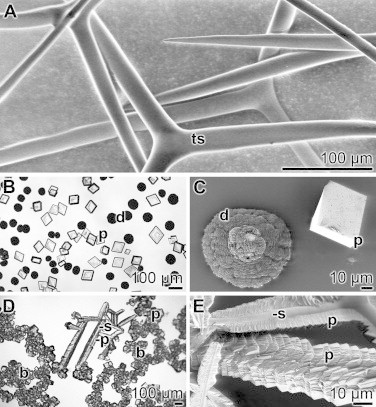
*S. raphanus* calcareous spicules and bio-calcite crystals formed *in vitro* with the CA enzyme. (**A**) Three-rayed spicules (ts); SEM image. (**B**) Ca-carbonate deposits [pat-like deposits (d), or prisms (p)] formed in a 50 mM CaCl_2_ solution under CO_2_. The reaction was performed in the presence of 3 units/mL CA for 5 h. LM image. (**C**) A pat-like deposit (d) and a prisms (p) formed under same conditions; SEM. (**D**) Association of the Ca-carbonate crystallites formed *in vitro* (in the presence of CA; 18 h). The Ca-carbonate prisms (p) are either arranged in ropes/bundles (b) or along a given *Sycon* spicule (s); LM. (**E**) The structure-guiding property of two sponge spicule (s) for calcitic prisms (p) along their surfaces is shown. The arrangement of the prisms is array-like and developed along opposing surfaces; SEM.

The enzymes CAes are found in all living taxa; they catalyze the rate-limiting reaction during Ca-carbonate mineral precipitation [15], i.e. the formation of bicarbonate, which is reversibly formed by a CA-mediated hydration of carbon dioxide [CO_2_]. In the present study we show that in the calcareous sponge spicular system for *Sycon raphanus* the homologous CA enzyme contributes essentially to the Ca-carbonate deposition by a considerable increase of its reaction velocity. The first experimental evidence that the CA is involved in calcareous spicule formation had been elaborated for *S. raphanus* [16]. The calcareous sponge *S. raphanus* reinforces its body both with diactines, two-rayed spicules, and with triactines, three-rayed spicules; the dimension of each ray varies between 100–170 μm in length and 6–10 μm in diameter (Fig. 1A).

## 2 Materials and methods

### 2.1 Animals

Specimens of *S. raphanus* (Porifera, Calcarea, Leucosolenida, Sycettidae) were collected in the Northern Adriatic Sea near Rovinj (Croatia). They were cultivated in the presence of 1 mM CaCl_2_. The spicules were isolated from the specimens with 0.5% [v/v] NaOCl [16].

### 2.2 Expression of carbonic anhydrase cDNA from S. raphanus

The complete cDNA (AMBL Accession No. HE610176), lacking the signal peptide as well as the transmembrane region, but comprising the complete carbonic anhydrase domain was expressed in *Escherichia coli* [16]. The bacterial cells were grown in Luria broth medium, containing 100 μM ZnSO_4_ [17]. The recombinant enzyme was purified by Ni-NTA agarose affinity chromatography. The specific activity was determined to be 2500 units/mg protein, by applying the Wilbur–Anderson assay [18,19].

### 2.3 Ca-carbonate precipitation assay

For preparation of Ca-carbonate precipitates, the ammonium carbonate diffusion method/“dessicator method” had been used [20,21]. The CO_2_ vapor was generated from a NH_4_HCO_3_ (Sigma; ≥99.0%) solution, placed in the lower compartment, which diffuses into the upper compartment of the dessicator, where a Petri dish with 5 mL of 50 mM CaCl_2_ (Sigma; ≥99.9%) was put. This solution was buffered with 10 mM HEPES [4-(2-hydroxyethyl)piperazine-1-ethanesulfonic acid] to pH 7.5. Routinely the assays were performed for up to 30 h at a temperature of 25 °C. Where indicated, 3 units/mL of the recombinant CA from *S. raphanus* was added to the CaCl_2_ solution. For the determination of the pH dependence of reaction, the assays were buffered with HEPES and 100 mM MES [2-(*N*-morpholino)ethanesulfonic acid] and then pH was adjusted. Where indicated the CA activity was inhibited by addition of 3 μM acetazolamide (A177 Sigma).

To follow up quantitatively the generation of Ca-carbonate, the free Ca^2+^ concentration in the CaCl_2_ solution was determined by EDTA titration [22]. Six parallel determinations were performed. The analyses given, including the hardness tests, were performed immediately after taking the samples.

### 2.4 Carbonic anhydrase esterase assay

The colorimetric assay using 4-nitrophenylacetate (NPA; Sigma) as a substrate was applied as described [23]. The assay (total volume of 750 μl) was composed of 15 mM Tris/SO_4_ buffer (pH 7.4), 3 mM 4-nitrophenylacetate, and 25 μl of recombinant enzyme sample (10 units of enzyme). The reaction was carried out at 22 °C for 3 min. The enzyme activity is given in mmoles·ml^−1^·min^−1^

### 2.5 Kinetic studies (Lineweaver–Burk plot)

The apparent Michaelis–Menten constants (*K*_m_) were calculated from Lineweaver–Burk plots [24].

### 2.6 Mechanical studies

Mechanical properties of the Ca-carbonate deposits formed were determined with a NanoTest Vantage system (Micro Materials Ltd., Wrexham, UK), equipped with a Berkovich diamond indenter, allowing continuous depth-sensing indentation [25,26]. Ten indents were performed for each measurement at 25 °C; maximum depth of an indentation was limited to 300 nm.

### 2.7 Microscopic inspections

Light microscopy (LM) was performed with a light digital microscope (VHX-600 Digital Microscope) from KEYENCE (Neu-Isenburg, Germany), equipped with a VH-Z25 zoom lens. For the scanning electron microscopic (SEM) analyses, a HITACHI SU 8000 (Hitachi High-Technologies Europe GmbH, Krefeld; Germany) was employed at low voltage (<1 kV; analysis of near-surface organic surfaces) [27].

### 2.8 Statistics

The results were statistically evaluated [28].

## 3 Results

### 3.1 Acceleration of bio-calcite deposition by CA

**Fig. 2:**
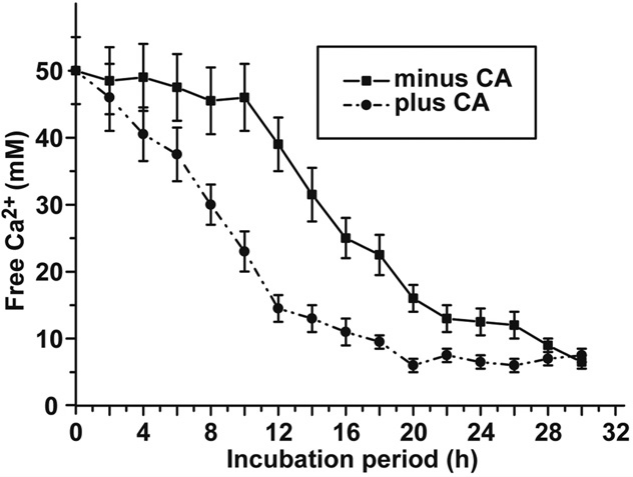
Acceleration of Ca-carbonate deposition in the ammonium carbonate diffusion assay by homologous CA. In the standard assay Ca-carbonate was allowed to precipitate after diffusion reaction of CO_2_ to a 50 mM CaCl_2_ solution. The concentration of free Ca^2+^ was determined by titration. The decrease in the concentration of free Ca^2+^ reflects the formation of insoluble Ca-carbonate. The reaction was performed in the absence (solid line) or presence of 3 units/mL of CA (broken line). After terminating the reaction, the samples (six parallel determinations have been performed for each reaction condition) were removed from the assay and subjected to EDTA titration. The means ± SD are given.

Using a starting concentration of 50 mM CaCl_2_, about 80% of this soluble salt was converted to insoluble Ca-carbonate after an incubation period of 24 h (at pH 7.5 and 25 °C) by using the ammonium carbonate diffusion assay. The mineralization process (based on the decrease of free Ca^2+^ concentration measured) started after an initial lag phase of 5 h (Fig. 2). Addition of the homologous recombinant CA (3 units/mL) significantly increased the reaction velocity and accelerated the mineralization process; after 5 h already 26% of the CaCl_2_ had been precipitated, in the presence of CO_2_, to Ca-carbonate (Fig. 2). An extent of 80% of precipitated Ca-carbonate was reached after 16 h.

The morphology of the Ca-carbonate crystals formed in the diffusion assay, irrespectively of the presence of the enzyme CA, changes with the progression of the incubation period. Initially, round shaped, pat-like precipitates are formed. In the absence of CA, those deposits are visualized during the first 12 h of incubation, while in the presence of the enzyme the aggregates appear already after 4–8 h (Fig. 1B and C). The sizes of the deposits formed in the absence of the CA are larger, 73±25 μm, than those developed in the presence of CA with 42±28 μm. Subsequently the round shaped pats remodel to crystal-like prisms morphology (Fig. 1D and E). That dominant morphology of the crystallites is characteristic for calcite crystals [29]. The crystallinic arrangement of these components has been established by X-ray diffraction. The energy-dispersive X-ray (EDX)-based elemental analyses revealed that the crystals are composed of the elements calcium, oxygen and carbon (to be published).

In the absence of *Sycon* spicules in the Ca-carbonate forming assay, the crystal-like prisms associate to each other and form rope-/bundle-like aggregates (Fig. 1D). However, after addition of *Sycon* spicules to the precipitation assay, the crystallites associate perfectly with their smaller planes along opposing surfaces of the spicule ray (Fig. 1D and E). The two arrays of crystals, the overgrowth, are facing each other along the spicules and leave the two remaining surface areas uncovered.

### 3.2 Dependence of CA-mediated calcite formation on temperature and pH

**Fig. 3:**
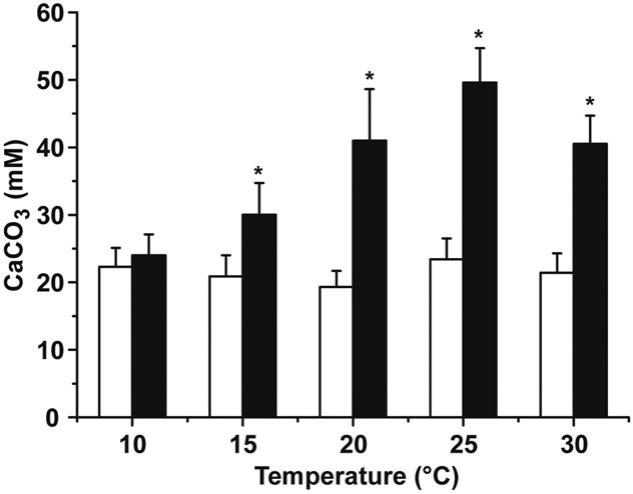
Dependence of Ca-carbonate formation at varying temperatures in the absence or presence of 3 units/mL CA. The mineralization assay had been performed under standard conditions (50 mM CaCl_2_; pH 7.5). After 15 h, the reaction was terminated and the concentration of free Ca^2+^ was determined by titration; the disappearance of Ca^2+^ reflects the appearance of Ca-carbonate precipitates; the results are given in mM of calculated Ca-carbonate formed. The experiments were performed in the absence of CA (white bars), or in the presence of 3 units/mL (black bars). Six parallel determinations have been performed for each condition, and were taken for EDTA titration. The means ± SD are given; *P < 0.05.

**Fig. 4:**
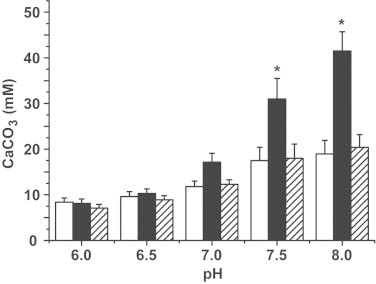
Effect of the changing pH conditions during the ammonium carbonate diffusion assay. The reaction was performed under standard conditions (50 mM CaCl_2_; 25 °C) in the absence (white bars) or presence of 3 units/mL CA (black bars). In one series of experiments the CA-driven reaction was performed in the presence of the CA-specific inhibitor acetazolamide (3 μM, hatched bar).

The extent of Ca-carbonate deposition at 10 °C is independent on the presence of CA and amounts to ≈24±3 mM·15 h^−1^ (pH 7.5) (Fig. 3). At higher incubation temperatures, the reaction velocity of CA-driven Ca-carbonate formation is significantly higher than that in the absence of CA. At 15 °C, the extent is 20.9±3.1 mM·15 h^−1^ in the absence of CA while it amounts to 30.4±4.6 mM·15 h^−1^ in the presence of 3 units/mL of CA; at 20–30 °C, the quantities of Ca-carbonate formed even doubles in the presence of CA, for example, at 25 °C the values are 49.6±5.1 (presence of CA) and 23.4±3.1 (absence of CA). In the absence of CA, the extent of Ca-carbonate precipitation does not change with the rising of temperature. Varying the pH value in the precipitation assay shows that in the absence of CA the precipitation of Ca-carbonate increases only slightly from pH 6.0 with 8.4±0.9 mM·15 h^−1^ (at 50 mM CaCl_2_) to 17.2±2.9 mM·15 h^−1^ at pH 8.0. In contrast, the CA-driven reaction velocity increases markedly from pH 6.0 with 8.1±1.0 mM·15 h^−1^ to 42.1±4.6 mM·15 h^−1^ (pH 8.0); Fig. 4. In a parallel study it was found that the enzyme CA-driven reaction has an optimum activity between pH 7.5 and 8.0 (to be published). To support the discovery that the Ca-carbonate deposition reaction is driven by the enzyme, a CA-specific inhibitor (3 μM acetazolamide [30]) had been added to the mineralization assay. These inhibition studies revealed that in the presence of 3 μM acetazolamide the amplification of the Ca-carbonate deposition reaction due to the presence of CA was almost completely abolished (Fig. 4).

### 3.3 Determination of the Michaelis–Menten constant for the CA-driven mineralization

**Fig. 5:**
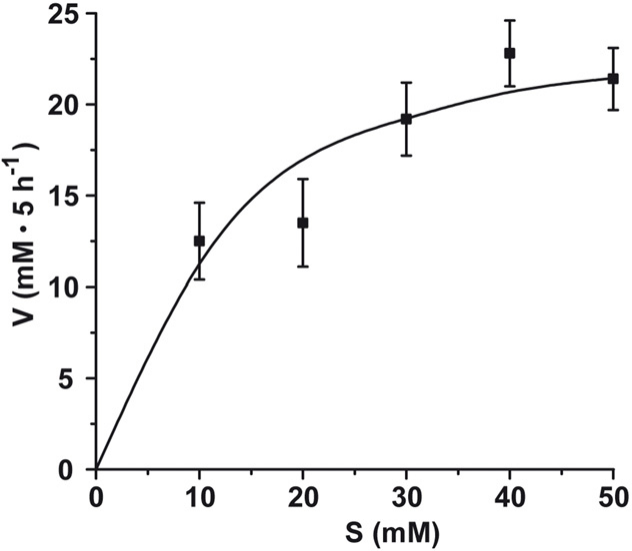
Enzymatic activity of CA during the Ca-carbonate mineralization CO_2_ diffusion assay. The substrate (CaCl_2_) was varied within the concentration range of 10 and 50 mM under standard assay conditions (3 units/mL of recombinant CA; 50 mM CaCl_2_, pH 7.5, 25 °C). The amount of Ca-carbonate formed, as calculated from the determined level of Ca^2+^ disappearance, is given in mM during 5 h incubation period.

The Ca-carbonate formation reaction follows substrate saturation kinetics. Under the assay conditions used here (50 mM CaCl_2_, pH 7.5, 25 °C), the linear increase of the reaction velocity is seen between 0 and 20 mM CaCl_2_, at higher concentrations the saturation level is approached (Fig. 5).

The CAes function both as a hydratase, formation of bicarbonate, and also as an esterase [31]. The Michaelis–Menten constants (*K*_m_) for both reactions are almost identical and vary around 5 mM for the hydratase (using CO_2_ as substrate [32]) and for the esterase (with the substrate 4-nitrophenylacetate [33]). The *K*_m_ constant for the sponge CA/esterase was determined by using the method of Lineweaver and Burk [34].

**Fig. 6:**
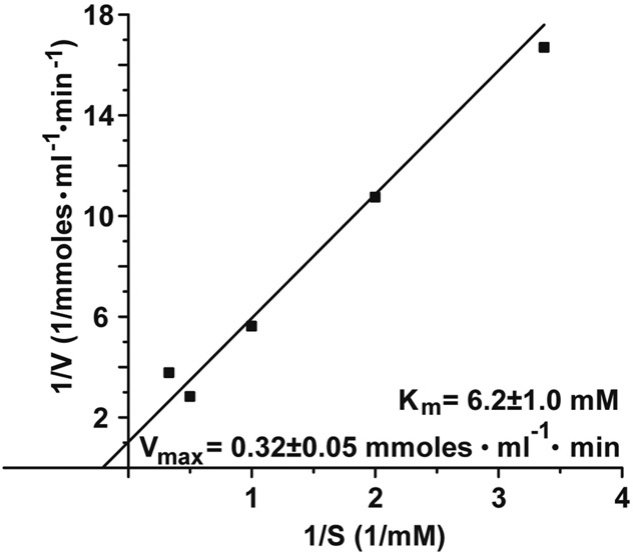
Lineweaver–Burk plot of 4-nitrophenylacetate by the recombinant sponge CA. The enzymatic activities were determined between 0.25 and 3 mM and the respective *K*_m_ value, as well as the *V*_max_ values were determined.

**Fig. 7:**
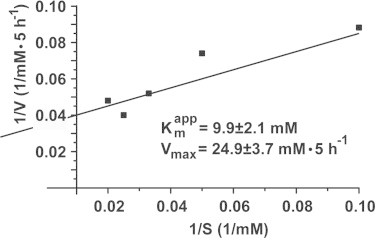
Affinity constant (Michaelis–Menten constant *K*_m_) for CA-driven Ca-carbonate mineralization using CO_2_ diffusion assay and CaCl_2_ as substrate. The substrate concentrations had been varied between 10 and 50 mM and the corresponding extent of Ca-carbonate precipitation had been determined on the basis of Ca^2+^ consumption in the standard assay. The apparent *K*_m_ value together with the *V*_max_ for Ca-carbonate synthesis was determined.

The apparent Michaelis–Menten constant for the sponge recombinant enzyme and using 4-nitrophenylacetate as esterase substrate was calculated from a Lineweaver–Burk plot [34]; Fig. 6. Varying the substrate concentration between 0.25 and 3 mM, a *K*_m_ constant of 6.2±1.0 mM was found, under a maximal reaction velocity of 0.32±0.05 mmoles·ml^−1^·min^−1^. Using the same approach, the Michaelis–Menten constant had been determined in the CO_2_ diffusion assay using 10 and 50 mM CaCl_2_ (Fig. 7). The Lineweaver–Burk plot was computed from which the apparent *K*_m_ value was determined (9.9±2.1 mM with respect to CaCl_2_) together with the corresponding *V*_max_ (24.9±3.7 mM Ca-carbonate formed during 5 h incubation period).

### 3.4 Abiogenic Ca-carbonate deposition, followed by enzymatic, biogenic Ca-carbonate synthesis

**Fig. 8:**
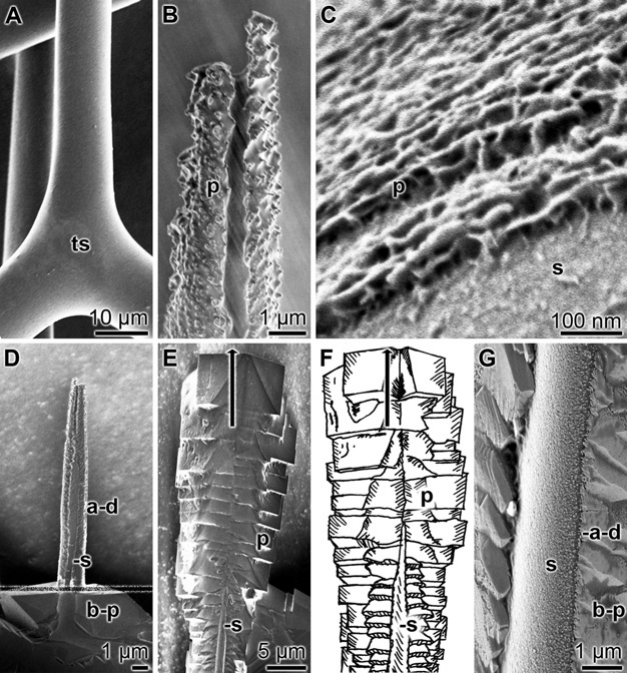
Stepwise abiogenic then biogenic Ca-carbonate synthesis proceeding on the surface of *Sycon* spicules; all are SEM images with the exception of the sketch (F). (**A**) Isolated sponge spicules. (**B** and **C**) Ca-carbonate deposits on the surface of spicules that had been incubated in the absence of CA for 10 h (50 mM CaCl_2_, pH 7.5, 25 °C). Small irregular crystals/prisms are labeled (p). An area of uncovered spicule surface is marked (s). (**D**) A stepwise incubation of the spicule (s) first for 5 h in the absence of the CA (upper part above the speckled line) and then for 5 h in the presence of 3 units/mL of CA (the spicule had been submerged in the enzyme assay below the speckled line) show the difference in the crystallites shape and form (abiogenically formed irregular deposits [a–d] versus biogenically produced calcitic prisms [b–p]). (**E**) Ca-carbonate prisms (p) formed around a spicule (s) during a 10 h incubation in the presence of CA. The prisms are orderly arranged. (**F**) Corresponding sketch highlighting the borders of the structures. The growth direction of the calcitic prisms is marked with an arrow. (**G**) A SEM image of a stepwise deposition (5 h abiogenically [a–d] followed by 5 h biogenically formed [b–p]) of Ca-carbonate prisms on the surface of a sponge spicule(s).

Cleaned *Sycon* spicules having a smooth surface (Fig. 8A) had been incubated in the CO_2_ diffusion chamber in the absence of CA for 10 h. During this period an approximately 40 nm thick layer of newly synthesized Ca-carbonate, composed of irregularly accumulated crystallites, are deposited onto the spicular surface (Fig. 8B and C). In contrast, if the spicules are incubated at first for 5 h in the absence of CA and then for additionally 5 h in the presence of CA the region of the spicules exposed to the enzyme solution become covered with orderly arranged Ca-carbonate deposits (Fig. 8D and E). During this process the new calcitic mantle around the spicule increases in size from about 40 nm to 7 μm. At a higher magnification the difference in morphology between the abiogenically formed irregular initial deposits and the biogenically formed regular prisms are becoming evident (Fig. 8G).

### 3.5 Mechanical properties of the Ca-carbonate deposits

The calcitic crystals formed in the CO_2_ diffusion assay were analyzed for their hardness using a Berkovich diamond indenter. After the recording of the load-displacement curves the corresponding Martens hardness had been determined. All the *in vitro* synthesized crystals show hardness values varying insignificantly between 4.2 ± 1.3 GPa and 4.7 ± 1.7 GPa. As a reference the hardness of the *Sycon* spicules had been determined with 5.3 ± 1.8 GPa.

## 4 Discussion

**Fig. 9:**
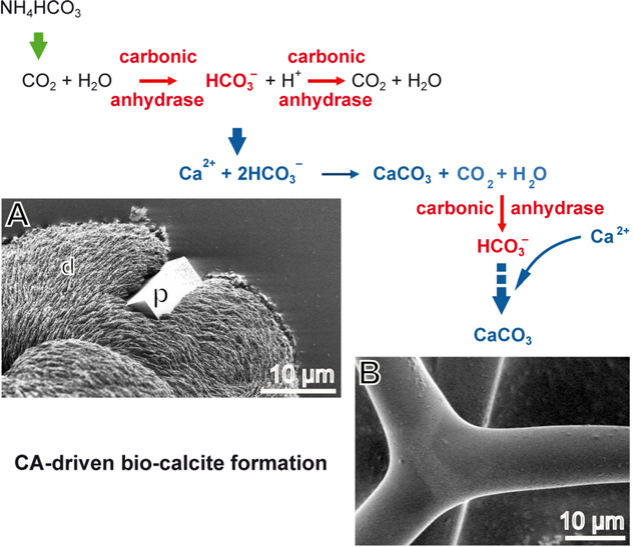
Relevance of the enzyme CA for the provision of bicarbonate, required for the biogenic synthesis of Ca-carbonate. Within the organism CA not only provides initially bicarbonate for the deposition of Ca-carbonate in the presence of Ca^2+^ but also speeds up this reaction via the removal of the liberate CO_2_ and water molecules after Ca-carbonate formation. It is concluded that the CA is a crucially important component for the formation of the (**A**) initially deposited pat-like deposits (d) which develop to prisms (p) that associate with existing calcareous structures. (**B**) The formation process from those crystals to a solid calcareous spicule remains to be studied.

The results reported here demonstrate that – as established for the siliceous sponge spicules [35,36] – the biomineralization process of the calcareous sponge spicules are also decisively driven enzymatically. Using the calcareous spicules from *S. raphanus* as an example, it is shown that the enzyme CA isolated from this animal and subsequently prepared in a recombinant way contributes essentially to the extent of the Ca-carbonate synthesis *in vitro*. As schematically outlined in Fig. 9, the enzyme CA is crucially important for the trapping of CO_2_/bicarbonate within an organism in general and a given cell in particular. The enzyme CA both provides bicarbonate to the respective transporters, and after translocation into the compartment, removes again bicarbonate from this site. During the synthesis of Ca-carbonate this enzyme again provides the required bicarbonate anion as a substrate for the formation of Ca-carbonate in the presence of Ca^2+^. Importantly, during this reaction again CO_2_ and water is released that served again as a substrate for the CA to initiate a further round of Ca-carbonate deposition. This implies that for one mole of Ca-carbonate two enzymatic CA-mediated steps are required to facilitate and accelerate the Ca-carbonate deposition. The experiments show an acceleration of the calcification process in the presence of the CA, following the kinetics of this enzyme with respect to the reaction temperature and pH as well as the Michaelis–Menten (*K*_m_) affinity constant. Remarkable is the finding that a rapid association of the newly formed crystals with the homologous spicules occurs in a highly ordered pattern.

In ongoing studies we could determine that the CA-driven Ca-carbonate formation *in vitro* starts with the synthesis of vaterite. This crystalline form is then converted to calcite. The conclusion comes from experiments using Fourier transform infrared spectrometry under determination of the characteristic absorption bands for vaterite and 745/744 cm^−1^ and calcite at 713/711 cm^−1^. These new data allow now the fabrication of calcitic structures along a calcareous “template” and opens new horizons for a biotechnological application of calcareous structures. In turn, the data reported also underscore again the utilization of sponge spicules as a molecular blueprint for the manufacturing of novel materials with hitherto unknown properties, like those recently shown for the fabrication of calcite spicules [37] along the structure-determining silicatein protein [38].
